# Subregions of the Rotator Cuff Muscles Present Distinct Anatomy, Biomechanics, and Function

**DOI:** 10.3390/sports12120349

**Published:** 2024-12-18

**Authors:** Emma Cavanaugh, Atenas Arcot Santillan, Kyosuke Hoshikawa, Hugo Giambini

**Affiliations:** 1 Department of Biomedical Engineering and Chemical Engineering, The University of Texas at San Antonio, San Antonio, TX 78249, USA; 2 Graduate School of Health Sciences, Yamagata Prefectural University of Health Sciences, Yamagata 990-2212, Japan

**Keywords:** rotator cuff, subregions, biomechanics, function

## Abstract

Shoulder and elbow injuries are prevalent among baseball players, particularly pitchers, who experience repetitive eccentric loading of the shoulder, leading to muscle damage and increased injury risk. Nearly 40% of shoulder injuries in baseball occur in pitchers, with many facing low rates of return to sport. The rotator cuff (RC) muscles—supraspinatus (SSP), infraspinatus (ISP), subscapularis (SSC), and teres minor (TMin)—are crucial for shoulder stability, movement, and force generation, particularly in overhead sports. Each RC muscle comprises subregions with distinct biomechanical properties, such as strength, moment arm behavior, and activation patterns. These differences allow for a finely tuned balance between joint stability and mobility. For example, the superior subregion of the ISP significantly contributes to external rotation, a function critical in sports like baseball that require precision and power. During pitching, the SSP, ISP, and SSC stabilize the glenohumeral joint through high activation during explosive phases, such as stride, arm cocking, and arm acceleration. Understanding these functional subregional differences is vital for diagnosing and managing shoulder pathologies like RC tears. Despite advancements, clinicians face challenges in predicting re-injury risks and determining return-to-play readiness for athletes with shoulder injuries. Integrating insights into subregional biomechanics with patient care could enhance outcomes. Tailored interventions—whether surgical or rehabilitative—targeting specific subregions could improve recovery times, reduce re-injury risks, and enable more personalized treatment plans. Such approaches are especially beneficial for athletes, older individuals, and those prone to RC injuries, promoting better long-term shoulder health and performance. The present work aims to highlight some of the research on these subregions and their differences, providing insights to enhance treatment approaches for shoulder injuries.

## 1. Introduction

Shoulder and elbow injuries are prevalent among baseball players, particularly pitchers. Although the overall injury rate in baseball is lower compared to contact sports like football, basketball, and soccer, overuse injuries remain a significant concern. Pitchers are disproportionally affected, with nearly 40% of shoulder injuries and 60% of elbow injuries occurring in this group [1]. Among adolescent pitchers, 15.7% of shoulder injuries and 25.3% of elbow injuries result in over three weeks of missed play [1]. Common conditions include rotator cuff (RC) tears, tendinopathy of the RC and biceps, shoulder impingement, labral lesions, and ulnar collateral ligament sprains.

Pitching involves repetitive eccentric loading of the shoulder and elbow muscles, leading to increased intra-compartmental pressure [2], muscle damage [3], altered muscle stiffness [4,5], and reduced contractile efficiency [3]. Improper mechanics that cause excessive joint loading or over-pitching without sufficient rest further exacerbate tissue degradation and overuse injuries [1,6].

The rotator cuff, comprising the supraspinatus (SSP), infraspinatus (ISP), teres minor (TMin), and subscapularis (SSC), is crucial for shoulder stability and a broad range of arm movements. These muscles originate from the scapula and attach to the humerus, forming a protective cuff around the glenohumeral joint [7]. Each muscle has specific roles: the SSP assists with arm abduction, while the ISP and TMin contribute to external rotation and adduction. Anatomically and biomechanically distinct subregions within the SSP, ISP, and SSC further refine their functional behaviors, emphasizing their heterogeneity.

During baseball pitching, the SSP, ISP, and SSC are particularly active, stabilizing the glenohumeral joint and supporting shoulder mechanics [8]. The RC muscles demonstrate high activation during explosive phases, with deltoid activity peaking during arm acceleration [8]. This intricate muscle coordination highlights the importance of understanding the subregional differences in anatomy and function, which is essential for tailoring surgical and rehabilitation interventions. The present work aims to highlight some of the research on these subregions and their differences, providing insights to enhance intervention approaches for shoulder pathologies in the setting of sports and aging. By addressing these intricacies, clinicians can optimize treatment outcomes, improve quality of life, and ensure athletes achieve a timely and safe return to sport.

### 1.1. Anatomy of the Rotator Cuff Muscles and Subregions

**Figure 1:**
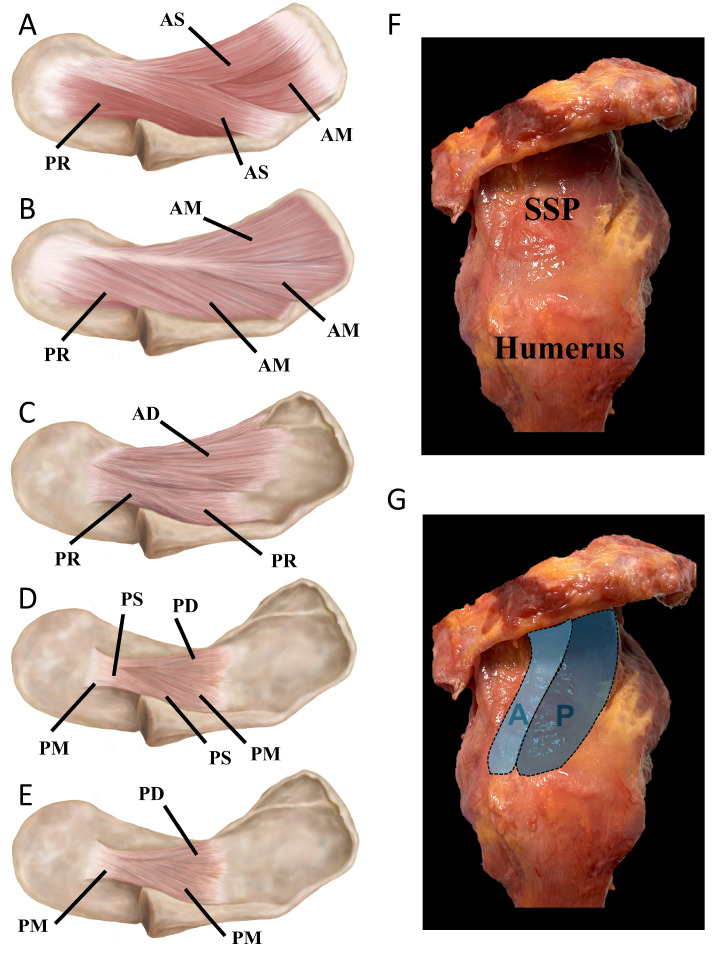
(**A**–**E**) Schematic of the supraspinatus (SSP) muscle showing the distinct subregions from a superior view. (**A**): Anterior–superficial region (AS), anterior–middle (AM), and posterior region (PR). (**B**): Removal of the AS subregion reveals the entire AM subregion with the intramuscular tendon. (**C**): Deep region of the anterior subregion (AD). (**D**): Posterior–superficial (PS), posterior–deep (PD), and posterior–middle (PM) subregions. (**E**): Removal of the PS reveals the entire PM and PD subregions. (**F**): Latero-superior view of a cadaveric specimen and of the SSP muscle. (**G**): Highlighted anterior (A) and posterior (P) SSP subregions (drawings by E. Cavanaugh and adapted from Kim et al. [10], with permission).

Supraspinatus muscle: While the architecture of the SSP muscle has been previously described as ranging from fusiform to circumpennate [9] or bipennate [10], a closer examination of the muscle and its tendon reveals a more complex architecture. Despite being relatively small, it accounts for 15% (40 ± 20 mL, as measured by water displacement and 3D models) of the total RC muscle volume [11]. Previous anatomical studies have divided the SSP muscle into anterior and posterior regions [12,13]. The anterior region of the SSP features a thick, tubular tendon with a small cross-sectional area (CSA) (26.4 ± 11.3 mm^2^), while the posterior region has a ‘strap-like’, flatter, and wider tendon with a larger CSA (31.2 ± 10.1 mm^2^) [12,13]. In contrast, the physiological CSA of the anterior region (140 ± 43 mm^2^) is larger compared to the posterior region (62 ± 25 mm^2^). In recent years, the SSP has been subdivided into six distinct subregions using a 3D model: (1) anterior–superficial, (2) anterior–middle, (3) anterior–deep, (4) posterior–superficial, (5) posterior–middle, and (6) posterior–deep (Figure 1) [10].

The lateral pennation angle of the anterior–deep subregion (74.1 ± 10.2°) is significantly greater than that of the anterior–middle subregion (51.4 ± 13.1°, *p* < 0.05) [10]. Similarly, the medial pennation angle of the anterior–deep subregion (16.8 ± 6.1°) is significantly greater than that of the anterior–superficial (11.4 ± 2.8°, *p* < 0.05) and anterior–middle subregions (9.8 ± 2.3°, *p* < 0.05). In contrast, the medial pennation angle of the posterior–superficial subregion (18.6 ± 7.6°) is significantly larger than that of the posterior–middle (11.3 ± 4.6°) and posterior–deep subregions (11.2 ± 3.6°, *p* < 0.05) [10].

The SSP muscle shows both loose and firm attachments to the footprint, characterized by less dense, spaced connective tissue, and tighter, denser connective tissue, respectively [14]. Specifically, the anterior and posterior tendons converge at the superior facet of the greater tuberosity of the humerus [15,16,17], and the posterior region, particularly the posterior–deep subregion, forms firm attachments to the joint capsule [14]. The innervation pattern of the suprascapular nerve to the distinct subregions of the SSP muscle is characterized by its division into two primary trunks: medial and lateral [18]. The medial trunk predominantly innervates the anterior subregions, while the lateral trunk primarily supplies the posterior subregions. Interestingly, in approximately half of the specimens analyzed in the study [18], the medial trunk also contributed additional innervation to the posterior region. These structural variations indicate potential functional differences between the superficial, deep, anterior, and posterior subregions.

**Figure 2:**
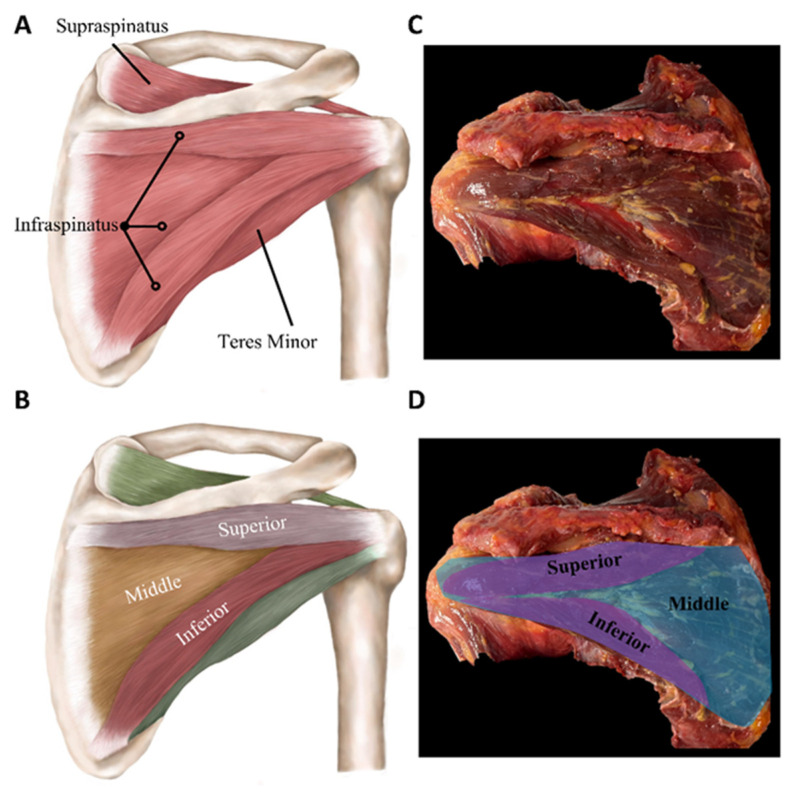
(**A**) Schematic of the infraspinatus (ISP) and teres minor (TMin) muscles. (**B**) Schematic of the superior, middle, and inferior subregions of the ISP muscle emphasizing the heterogeneity in muscle fiber direction and tendon attachment areas. (**C**) Cadaveric ISP muscle and (**D**) highlighted subregions (drawings by E. Cavanaugh).

Infraspinatus muscle: Among the RC muscles, the ISP muscle, trapezoidal in shape, has the second-largest anatomic footprint on the humerus [14]. The ISP originates from the infraspinous fossa beneath the scapular spine, with muscle fibers ascending superolaterally and intertwining with its tendon [15]. As the tendon crosses the bare area of the scapula, it broadens into a flattened structure before attaching to the posterosuperior aspect of the greater tuberosity [15,16]. The ISP has been described as having two bellies connected by a central groove: the superior belly associates with the ISP muscle, while the inferior belly represents the TMin muscle [19]. The ISP is composed of three distinct muscular subregions, (1) superior, (2) middle, and (3) inferior, each housed in a thin fascial compartment but all located deep to the posterior scapular fascia (Figure 2) [19]. Similar to the posterior subregion of the SSP muscle, the middle subregion of the ISP muscle inserts and forms firm attachments to the capsule [14]. Minagawa et al. showed that the SSP and ISP tendons interdigitate in the posterior half region of the SSP tendon [20], and Yuri et al. expanded on this finding by showing that the middle subregion of the ISP tendon and posterior–deep subregion of the SSP tendon evenly occupied the capsular attachment area [14]. A first-order suprascapular nerve, representing the initial division from the main nerve stem, is observed in 91.6% of the superior, 100% of the middle, and 70.8% of the inferior subregions, and in 62.5% of the specimens assessed, it was consistently present across all three subregions of the same infraspinatus muscle [19].

Teres minor muscle: While the morphological and functional aspects of the TMin muscle have garnered clinical interest, there have been limited investigations into its anatomical details. The TMin muscle is often grouped with the ISP, resulting in a scarcity of literature specifically describing the muscle’s structure and attachment onto the humerus. Research has shown that the insertion of the TMin resembles an oblong shape [21], originating from the infraspinous fossa and comprising two distinct and independent muscular bundles: the upper and lower portions [22]. The upper portion originates from the lateral edge of the scapula as a tendinous attachment and inserts into the posterior region of the greater tuberosity in a round shape. The lower portion emerges from the fascia between the TMin and ISP, as well as the inferior surface of the lateral edge of the scapula, serving as a muscular attachment that inserts into the neck of the humerus [22].

**Figure 3:**
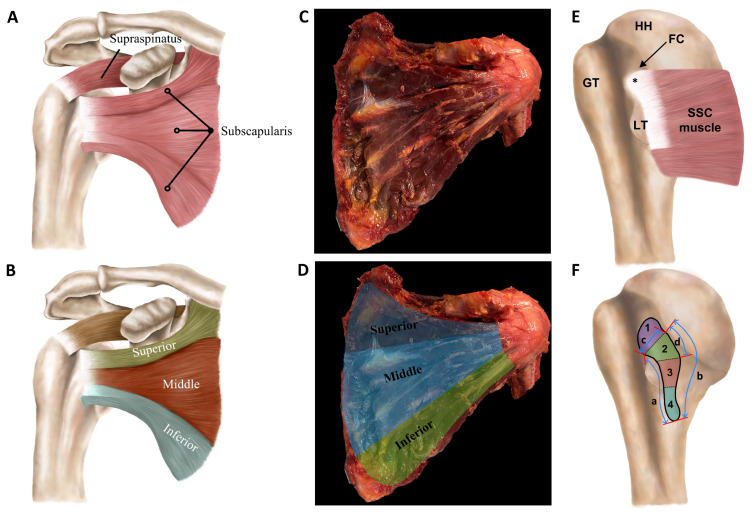
(**A**) Schematic of the subscapularis (SSC) muscle. (**B**) Superior, middle, and inferior subregions of the SSC muscle are depicted to emphasize the heterogeneity in muscle fiber direction and tendon attachment areas. (**C**) Cadaveric SSC muscle and (**D**) highlighted subregions. (**E**) Insertion area. The superior portion of the intramuscular tendon attaches to the uppermost section of the lesser tuberosity (LT), while the remainder of the tendon anchors to the anteromedial part of the LT. The uppermost attachment, along with the lateral section of the upper/cranial part of the intramuscular tendon and the tendinous slip (*), closely contact the inferior side of the long head of the biceps tendon at its corner. (**F**) Footprint area: 1: attachment area of tendinous slip; 2: insertion of superior-most part of intramuscular tendon; 3: tendinous insertion; and 4: muscular insertion. Footprint dimensions: a: lateral margin; b: medial margin; c: superior-most lateral margin; and d: superior-most medial margin (HH, humeral head; FC, fovea capitis of humerus; GT, greater tuberosity) (drawings by E. Cavanaugh and adapted from Arai et al. [25], with permission).

Subscapularis muscle: As the largest muscle among the four comprising the RC, the SSC accounts for 40% (106 ± 45 mL, as measured by water displacement and 3D models) of the total RC muscle volume [11]. The SCC muscle occupies the majority of the subscapular fossa and serves as the sole internal rotator due to its anterior attachment on the scapula (Figure 3) [23]. Unlike the ISP and SSP muscles, which attach to the greater tuberosity, the SSC muscle inserts onto the lesser tuberosity [23]. Its tendon exhibits a broader superior insertion, narrowing inferiorly to form a trapezoidal shape, with the tendinous portion accounting for approximately 60% of the footprint, while the muscular portion comprises the remaining 40%, featuring a direct attachment of muscle fibers to bone [24]. The SSC muscle displays a complex insertion pattern, consisting of a tendinous insertion for the superior two-thirds and a muscular insertion for the inferior one-third, where the muscle attaches almost directly to the humerus via a thin membranous structure [25]. The fibers run laterally from their origin, with the superior-most insertion extending widely into the upper margin of the lesser tuberosity, while the remainder inserts into the anteromedial portion of the lesser tuberosity, accompanied by a thin tendinous slip attaching to the fovea capitis of the humerus [25]. There is an ongoing debate regarding the insertion of the SSC tendon, with research investigating whether it attaches solely to the lesser tuberosity or extends medially and laterally beyond this point.

Ward et al. investigated the architectural properties of the SSC subregions, finding that the superior subregion has shorter muscle fiber lengths than the inferior subregion (*p* = 0.029) [26]. Cho et al. examined the innervation patterns of the upper subscapular, lower subscapular, thoracodorsal, and axillary nerves in the SSC subregions [27]. They found that the superficial branches of the upper subscapular nerve are primarily distributed in the superior and middle portions, while the deep branches of both the upper and lower subscapular nerves are distributed in the inferior portion. Additionally, the thoracodorsal and axillary nerves innervate the inferior portion [27]. These findings offer a more comprehensive understanding of SSC muscle structure, which can enhance knowledge of its function and help inform more effective rehabilitation strategies for RC injuries.

### 1.2. Biomechanical Properties of the Rotator Cuff Subregions

#### 1.2.1. Moment Arms

A moment arm is defined as the perpendicular distance between a muscle’s line of action and the joint’s center of rotation, reflecting the muscle’s capacity to generate torque [28]. It indicates the muscle’s leverage around a joint, determining whether the muscle shortens or lengthens during joint movement. Table 1 summarizes some of the previously assessed outcomes.

Infraspinatus moment arms: Positive moment arms indicative of external rotation moments are observed in the ISP muscle when the humerus is in both neutral and abducted positions [29]. During external rotation in the neutral position, moment arms for the superior, middle, and inferior subregions range from 19.1 ± 3.8 mm to 21.5 ± 4.1 mm, which are larger than the internal rotation moment arms, ranging from 14.0 ± 3.6 mm to 17.3 ± 4.4 mm. Similarly, when the humerus is abducted, larger moment arm values are observed during external rotation (10.8 ± 5.6 mm to 18.1 ± 6.6 mm) compared to internal rotation (8.7 ± 5.3 mm to 12.0 ± 7.5 mm). In the abducted position, when the humerus is rotated from an internal to an external position, the middle and inferior subregions exhibit larger moment arms compared to the superior subregion (10 ± 6.3 mm, 12 ± 7.5 mm, 8.7 ± 5.3 mm for internal rotation, and 15.9 ± 5.6 mm, 18.1 ± 6.6 mm, 10.8 ± 5.6 mm for external rotation, respectively). When evaluating external and internal rotations separately, the moment arms of the middle subregion are comparable to those of the superior subregion, while the inferior subregion consistently differs. Overall, the superior subregion exhibits smaller moment arms across all motions compared to the inferior and middle subregions [29]. In contrast, during flexion, all ISP subregions display positive and nearly constant moment arms [30]. These observations are further supported by Ackland et al. who showed that the inferior subregion of the ISP was the greatest external rotator with moment arms peaking at 28.3 mm during abduction at 120° and 23.3 mm during 30° flexion [31].

Supraspinatus moment arms: In a neutral position, both the anterior–superficial and anterior–middle subregions of the SSP muscle exhibit lower moment arms compared to the posterior–deep subregion during internal rotation (−0.6 ± 2.4 mm, −1.3 ± 3.2 mm, and 9.2 ± 5.1 mm, respectively) [29]. A similar trend is observed during external rotation, where the posterior–deep subregion demonstrates larger moment arms than both the anterior–superficial and anterior–middle subregions (10.6 ± 6.2 mm, 1.8 ± 3.4 mm, and 1.5 ± 3.1 mm, respectively). In the abducted position, moment arms for the anterior–superficial (2.9 ± 2.2 mm and 2.1 ± 1.0 mm) and anterior–middle (3.9 ± 7.3 mm and 3.6 ± 9.2 mm) subregions are slightly higher, but still lower than those of the posterior–deep subregion (6.4 ± 5.9 mm and 8.3 ± 8.5 mm). The posterior–deep subregion, with positive moment arms during shoulder rotation in both neutral and abducted positions, functions similarly to the ISP muscle as an external rotator. Conversely, the anterior–superficial and anterior–middle subregions display biphasic behavior during rotation in a neutral position, acting as slight internal rotators during internal rotation and external rotators during external rotation. However, when the humerus is abducted, these subregions act purely as external rotators [29]. Similar outcomes have been reported supporting the role of the posterior subregion as the largest external rotator during abduction [28,31,32,33,34,35]. During flexion, the anterior–superficial and anterior–middle subregions exhibit positive but decreasing moment arms as flexion angles increase, acting as flexors and internal rotators [30]. The posterior deep subregion maintains positive moment arms up to 65°, transitioning to negative moment arms thereafter, thus functioning as extensors [30]. Combined anterior and posterior flexion outcomes are shown in Table 1.

Subscapularis moment arms: Negative moment arms are observed during rotation with the shoulder in neutral [29], abducted [29,31], or flexed positions [30,31], representative of internal rotator and depressor behaviors. Ackland et al. showed that the inferior SSC subregion was the largest internal rotator during abduction and flexion [31]. The study showed the inferior SSC subregion to present peak moment arms of 24.4 and 27.0 mm at 30° of abduction and flexion, respectively [31]. Those previous observations are supported by Yuri et al., who demonstrated that during external and internal rotation with the humerus abducted, the inferior subregion had the largest moment arms compared to the middle and superior subregions (−18.5 ± 8.1 mm, −14.3 ± 8.7 mm, and −7.1 ± 7.6 mm, respectively) [29]. While the inferior subregion presents negative moment arms with increasing angles throughout flexion, the superior and middle subregions show positive, but decreasing, moment arms with increasing angles [30].

Key Insights: The distinct biomechanical behaviors of muscle subregions reflect their specialized roles: the posterior–deep supraspinatus and inferior infraspinatus behave as external rotators, while the inferior subscapularis serves as a powerful internal rotator and depressor. These variations, dependent on joint position, demonstrate the intricate interplay between muscle architecture and function, critical for movement, rehabilitation, and surgical planning.

**Table 1:** Moment arms (mm) of the RC muscles and subregions with various arm positions {mean (SD) [range]} [29,31].

Muscle		Internal Rotation	External Rotation	Internal + External Rotation
Infraspinatus (S: superior; M: middle; I: inferior)				
Neutral	S	14.0 (3.6)	19.1 (3.8)	17.5 (4.4)
	M	15.5 (5.0)	20.6 (5.6)	19.1 (5.8)
	I	17.3 (4.4)	21.5 (4.1)	20.4 (4.9)
60° abduction	S	8.7 (5.3) **^c,e^**	10.8 (5.6) **^d,f^**	10.3 (5.5) **^a,b,e^**
	M	10.0 (6.3)	15.9 (5.6)	13.5 (6.5)
	I	12.0 (7.5)	18.1 (6.6)	15.6 (7.5)
Flexion (30°)	S			13.0 (2.8) [9.5 to 18.9]
	I			15.8 (3.9) [10.3 to 20.9]
**^a^**: *p* = 0.042 compared to M; **^b^**: *p* < 0.001 compared to I; **^c^**: *p* = 0.029 compared to I; **^d^**: *p* = 0.018 compared to I; **^e^**: *p* < 0.001 compared to TMin; **^f^**: *p* = 0.017 compared to TMin.				
Teres Minor				
Neutral		15.0 (4.3)	21.8 (4.6)	19.6 (5.3)
60° abduction		14.2 (7.8)	19.7 (3.5)	17.5 (6.1)
Flexion (30°)				16.4 (4.3) [7.3 to 20.5]
Supraspinatus (A: anterior; P: posterior; AS: anterior–superficial; AM: anterior–middle; PD: posterior–deep)				
Neutral	AS	−0.6 (2.4) **^a^**	1.8 (3.4) **^b^**	0.7 (3.2) **^a^**
	AM	−1.3 (3.2) **^a^**	1.5 (3.1) **^a^**	0.1 (3.3) **^a^**
	PD	9.2 (5.1)	10.6 (6.2)	10.5 (6.3)
60° abduction	AS	2.9 (2.2)	2.1 (1.0) **^c^**	2.4 (1.6) **^a^**
	AM	3.9 (7.3)	3.6 (9.2) **^d^**	4.0 (8.2) **^a^**
	PD	6.4 (5.9)	8.3 (8.5)	7.5 (7.3)
Flexion (30°)	A			−8.3 (2.2) [−11.3 to −4.0]
	P			−0.9 (0.9) [−5.8 to 5.4]
**^a^**: *p* < 0.001 compared to PD; **^b^**: *p* = 0.003 compared to PD; **^c^**: *p* = 0.033 compared to PD; **^d^**: *p* = 0.013 compared to PD.				
Subscapularis (S: superior; M: middle; I: inferior)				
Neutral	S	−13.2 (7.4)	−17.5 (4.5)	−16.2 (6.5) **^a^**
	M	−16.0 (7.1)	−22.2 (6.6)	−20.0 (7.6)
	I	−12.3 (6.7)	−21.0 (6.1)	−18.1 (7.5)
60° abduction	S	−4.2 (6.0)	−9.6 (7.8)	−7.1 (7.6)
	M	−10.9 (8.2)	−16.6 (8.1)	−14.3 (8.7) **^b^**
	I	−13.6 (9.1) **^c^**	−22.0 (5.5) **^d^**	−18.5 (8.1) **^b^**
Flexion (30°)	S			−15.5 (1.8) [−17.5 to −13.2]
	M			−17.6 (1.7) [−20.0 to −15.2]
	I			−19.4 (3.8) [−23.5 to −13.0]
**^a^**: *p* = 0.049 compared to M; **^b^**: *p* < 0.001 compared to S; **^c^**: *p* = 0.002 compared to S; **^d^**: *p* < 0.001 compared to S.				

#### 1.2.2. Tensile Properties

The musculotendinous component consists of the muscle itself, the tendon that interdigitates with the muscle, and the enthesis, where the tendon inserts into the bone [36]. Tendons can be viewed as unidirectional, fiber-reinforced composites that connect muscles to bones and transmit forces generated by the muscle into the joints [37,38]. The concept of dividing the RC muscles and tendons into distinct subregions, each with specific mechanical properties and functions, offers a valuable perspective for understanding shoulder biomechanics. Numerous studies have investigated the mechanical properties of RC tendons under tension and failure [39,40], as well as the effects of cyclic loading on repair techniques [41,42,43,44,45]. Importantly, there are few studies focusing on the failure properties of RC tendons as they relate to their various subregions [39,40,46,47,48].

A tendon, or its insertion into bone, may rupture under tensile loads that exceed its ultimate strength. Tensile loads of the SSP, ISP, and SSC muscles has been reported for the whole muscle and its individual subregions. Table 2 summarizes previously assessed outcomes. Specifically, the ultimate tensile loads for the anterior and posterior tendon subregions of the SSP muscle is recorded at 779 ± 219 N and 335 ± 164 N, respectively [40]. For the ISP tendon, structural and material properties were assessed in various subregions–superior, mid-superior, mid-inferior, and inferior—in both a hanging-arm position and at 60° of glenohumeral abduction [39]. The ultimate tensile load of the mid-superior (662.4 ± 223.4 N) and inferior (716.6 ± 252.7 N) tendon subregions is significantly greater than that of the mid-inferior subregion (330.8 ± 205.8 N) in the hanging-arm position. At 60° of abduction, the mid-superior strip (696.8 ± 258.1 N) demonstrates significantly higher ultimate tensile loads than the superior (424.2 ± 154.4 N), mid-inferior (301.6 ± 168.5 N), and inferior tendon subregions (406.9 ± 239.0 N) [39]. Glenohumeral abduction resulted in differences in elastic modulus across the tendon subregions, while structural properties showed no significant variation.

Mechanical properties of the SSC muscle subregions—superior, mid-superior, mid-inferior, and inferior—were obtained in a hanging-arm position and at 60° of abduction [46]. The mean ultimate tensile loads for the SSC subregions were 623 N, 706 N, 454 N, and 75 N in the hanging-arm position, which, while not statistically significant, are greater than those at 60° of abduction (478 N, 598 N, 400 N, and 30 N, respectively). Furthermore, stiffness varied between tendon subregions based on arm position; the stiffness of the inferior tendon subregion was significantly higher in the hanging-arm position (27.4 ± 18 N/mm) compared to 60° of abduction (9.5 ± 6.0 N/mm). Conversely, the superior tendon subregion presented greater stiffness at 60° of abduction (209 ± 61 N/mm) than in the hanging-arm position (147 ± 32 N/mm). Given the variety of tasks and shoulder positions encountered in daily activities, understanding the effects and properties observed in both whole tendons and their subregions is crucial.

Key Insights: The mechanical properties of RC tendon subregions vary significantly based on shoulder position, with distinct tensile loads and stiffness reflecting their specialized roles during movement. This positional dependence demonstrates the importance of understanding subregional behavior for improving clinical intervention strategies.

### 1.3. Functional Properties of the Rotator Cuff Subregions

While there are various imaging techniques (i.e., magnetic resonance imaging [49] and ultrasonography [50]) that are used to assess the presence/absence of certain pathologies at the shoulder and clinical measures (i.e., range of motion and strength measures) that provide the global assessment of the soft tissues around the joint, there are currently no clinical methods being implemented that allow for the intrinsic evaluation of properties of the specific muscles. Shear wave elastography (SWE) is an ultrasound technique that has been previously used to obtain quantitative estimations of tissue shear modulus, as a surrogate for tissue stiffness, of various muscles and their subregions [51,52,53,54,55,56].

**Figure 4:**
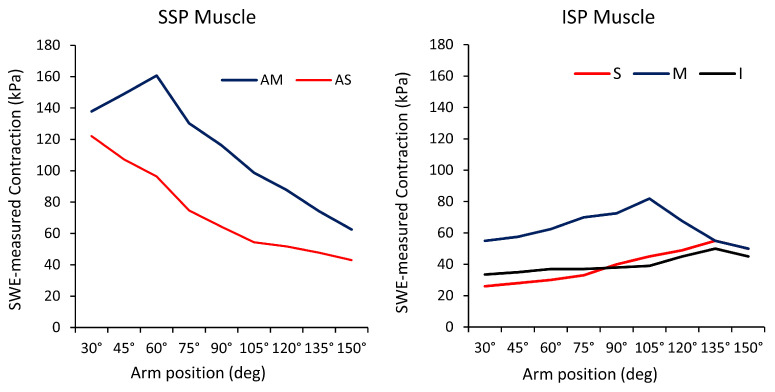
Mean SWE-measured contraction outcomes for the anterior–middle (AM) and anterior–superficial (AS) subregions of the SSP muscle [58], and of the superior (S), middle (M), and inferior (I) subregions of the ISP muscle during scaption [57]. Adapted from Hoshikawa et al. [58], with permission. Adapted from Hoshikawa et al. [57] under a Creative Commons Attribution International License (http://creativecommons.org/licenses/by/4.0/); accessed on 5 December 2024.

Shoulder Scaption: Scapular plane abduction, also known as scaption, is an essential movement for upper limb activities in daily life. Hoshikawa et al. investigated the distinct functional roles of the ISP muscle subregions during shoulder scaption in healthy subjects using SWE [57]. The study found that muscle activity, defined as the difference in SWE-measured stiffness between rest and contraction, was greatest in the middle subregion throughout scaption (from 30° to 150° in 15° intervals), peaking at 105° (52.2 ± 10.8 kPa, *p* < 0.001). At the end range of scaption, the inferior and superior subregions presented higher activity than at the initial range of motion, peaking at 135° (23.0 ± 12.0 kPa, *p* <0.001, and 32.9 ± 13.8 kPa, *p* < 0.001, respectively) [57]. Heterogeneous activity patterns were also observed among the subregions of the SSP muscle and the middle subregion of the deltoid muscle [58]. During the initial phase of scaption, activity was primarily noted in the anterior–superficial subregion of the SSP muscle, peaking at 30° (102 ± 27.4 kPa, *p* < 0.029). In the early mid-range (30–60°), the anterior–middle subregion of the SSP muscle became active, reaching peak values of 146.2 ± 26.6 kPa (*p* < 0.002), while increased activity in the late mid-range was noted in the middle region of the deltoid muscle (142.0 ± 25.9 kPa, *p* < 0.007) [58]. Figure 4 shows a summary of subregional outcomes using elastography.

Wickham et al. used electromyography (EMG) to explore the functional differences between the upper and lower regions of the SSC muscle during shoulder abduction [59]. They observed that the lower region exhibited a significantly faster onset (*p* = 0.018, effect size = 1.40) and higher activity than the upper region (*p* < 0.001, effect size = 1.43), particularly in the mid-range of abduction. Similarly, Alenabi et al. analyzed the activity of SSP and ISP subregions during isometric arm elevation under maximal and submaximal (50% MVF) resistance using EMG [60]. Their results indicated that the anterior region of the SSP muscle was significantly more active during abduction and scaption compared to flexion, especially at higher elevation angles. In contrast, the posterior region exhibited consistent activation across all elevation planes. Notably, the SSP anterior region’s greater activity during scaption at maximal and submaximal resistance levels contrasts with Hoshikawa et al.’s findings under unloaded conditions [58], where the SSP muscle’s anterior subregions showed peak activity in the initial range. This discrepancy suggests the importance of further research into the effects of varying resistance levels on muscle activation.

Shoulder Rotation: The RC muscles are crucial for both internal and external rotation of the shoulder. Internal rotation is primarily facilitated by the SSC muscle, while external rotation is mainly supported by the SSP and ISP muscles. Yuri et al. utilized real-time tissue elastography (RTE) to assess muscle activity, defined as the difference in strain ratio between rest and contraction, in the SSP subregions of healthy individuals during internal and external rotation at 90° abduction [61]. They found that the activities of the anterior–superficial, anterior–middle, anterior–deep, and posterior–deep subregions were highest at the neutral position (*p* < 0.05), and that the activities during external rotation were significantly higher than during internal rotation (*p* < 0.05), except that of the anterior–deep subregion. In contrast, the activity of the posterior–superficial and posterior–middle subregions showed consistently low activity throughout rotation. Similarly, Yuri et al. utilized SWE to explore the passive and active properties of the ISP and TMin muscles in healthy individuals during external rotation at 0° of abduction, revealing heterogeneity within the ISP subregions [62]. Specifically, the inferior subregion consistently presented decreased outcomes with increasing rotation angles (*p* < 0.001), while the superior subregion maintained constant values throughout active external rotation, except at 60° where a slight increase was observed. The middle subregion showed an initial increase in outcomes with external rotation angles up to 30°, followed by a decrease at 60° (*p* < 0.001). Similarly, Langenderfer et al. reported that the inferior subregion of the ISP muscle generated peak force at the initial range of rotation, while the superior subregion exhibited consistent behavior, and the middle subregion peaked at mid-range motion [63]. Boettcher et al. examined the SSC muscle, finding that during internal rotation, the activity of the superior and inferior subregions was significantly higher than during external rotation (*p* < 0.001) [64]. Furthermore, they observed that the inferior subregion’s activity was higher than that of the SSP and ISP muscles (*p* < 0.02 and *p* < 0.001, respectively), while the superior subregion’s activity was higher than that of the ISP muscle (*p* < 0.001).

**Figure 5:**
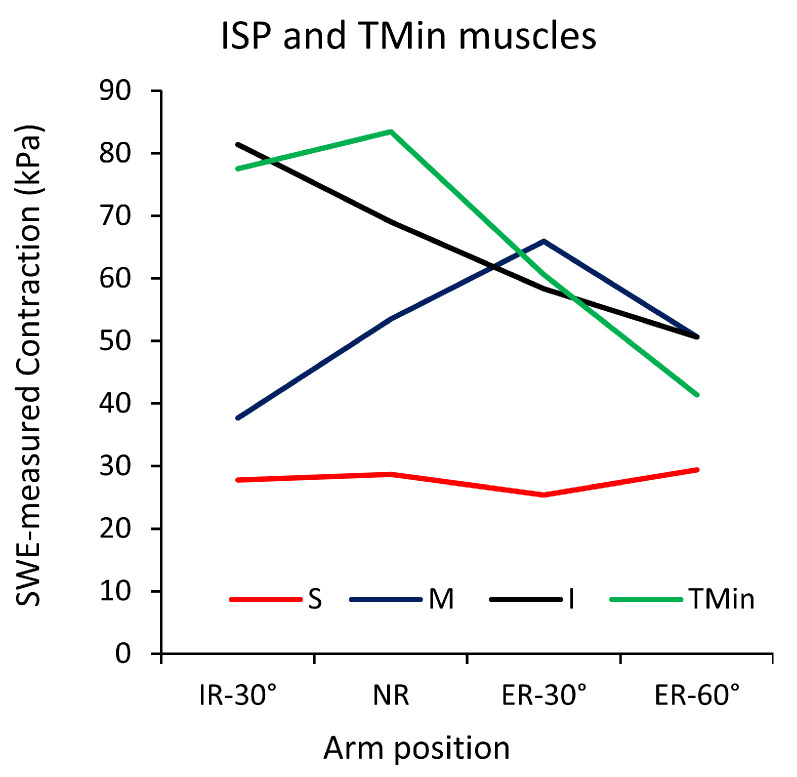
Mean SWE-measured contraction outcomes for the superior (S), middle (M), and inferior (I) subregions of the ISP muscle and for the TMin muscle during external rotation. IR, internal rotation; NR, neutral; ER, external rotation. Adapted from Yuri et al. [62], with permission.

Whittaker et al. examined the activation patterns of the SSP and ISP subregions during external rotation exercises at various shoulder positions to optimize rehabilitation [65]. Their findings showed that the anterior and posterior regions of the SSP exhibited the highest activity during external rotation at 90° of abduction, with maximum intensity activations of 61.6% ± 3.1% and 56.2% ± 4.1% of maximum voluntary isometric contraction (MVIC), respectively. The superior subregion of the ISP showed greater activity at 0° of elevation (50.9% ± 5.7% MVIC) compared to 30° of scaption (37.4% ± 3.9% MVIC) at maximal intensity. Conversely, the inferior subregion demonstrated higher activity at 90° of scaption during both maximal and submaximal intensities (59.8% ± 2.8% and 29.4% ± 1.9% MVIC, respectively) compared to 0° of elevation. Figure 5 shows a summary of subregional outcomes during rotation using SWE.

**Figure 6:**
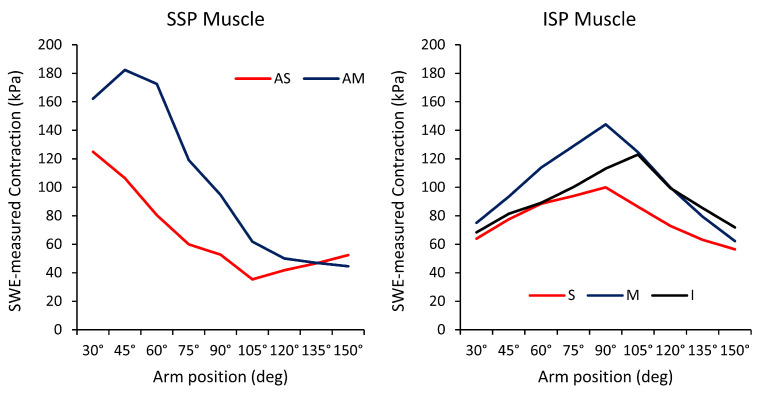
Mean SWE-measured contraction outcomes for the anterior–middle (AM) and anterior–superficial (AS) subregions of the SSP muscle, and of the superior (S), middle (M), and inferior (I) subregions of the ISP muscle during flexion. Adapted from Hoshikawa et al. [67], with permission.

Shoulder Flexion and Extension: The RC muscles play a vital role in maintaining dynamic stability of the shoulder joint during flexion, preventing abnormal translation of the humeral head. Research indicates that the SSP and ISP muscles exhibit higher levels of activity during flexion compared to extension, with both muscles showing similar activation patterns during flexion [66]. In contrast, the SSC muscle demonstrates greater activation during extension than during flexion [66]. Notably, functional differences exist among the anatomical subregions of the RC muscles during flexion. Hoshikawa et al. utilized SWE to evaluate the activity of the individual subregions of the SSP and ISP muscles during isometric contraction in a neutral position and at 15° intervals from 30° to 150° during flexion [67]. The activity of the anterior–middle subregion of the SSP muscle increased between 30° and 45°, reaching a peak of 182.4 ± 32.1 kPa (*p* < 0.001). Conversely, the anterior–superficial subregion exhibited the highest values at 30° (125.0 ± 20.6 kPa, *p* < 0.019), with a linear decrease up to 105°. In the ISP muscle, outcomes in the superior, middle, and inferior subregions followed a mountain-shaped trend, peaking at 90° for the superior (99.9 ± 23.5 kPa, *p* < 0.013) and middle subregions (144.2 ± 11.2 kPa, *p* < 0.013), and at 105° for the inferior subregion (122.9 ± 27.9 kPa, *p* < 0.007). Figure 6 shows a summary of subregional outcomes using elastography.

Alenabi et al. assessed the activity of the SSP and ISP subregions during isometric arm elevation under maximal and submaximal resistance conditions using EMG [60]. Their findings showed that the ISP superior and inferior regions were more active during flexion than abduction, consistent with results from studies utilizing SWE [57,67]. Wickhan et al. investigated the functional differences between the upper and lower regions of the SCC muscle during flexion using EMG [59]. They found that the lower region exhibited significantly higher activity than the upper region (*p* < 0.001) and the upper region showed low activity throughout flexion motion.

Key Insights: Shear wave elastography (SWE) and electromyography (EMG) have provided insights into the functional variability of RC muscle subregions across different shoulder motions. During scaption, subregions of the ISP and SSP muscles show varying activity patterns, with the middle subregion of the ISP being most active at mid-range angles and the anterior subregion of the SSP peaking early in motion. In rotation, ISP subregions display stiffness changes tied to specific angles, while the SSC shows greater activation during internal rotation. For flexion and extension, subregions of SSP, ISP, and SSC demonstrate distinct stiffness and activity profiles, highlighting differing roles in dynamic shoulder stability. These findings emphasize the biomechanical diversity within RC muscles, influenced by motion type, angle, and load conditions.

## 2. Conclusions

The rotator cuff (RC) muscles, comprising the supraspinatus (SSP), infraspinatus (ISP), subscapularis (SSC), and teres minor (TMin), are essential for shoulder function and joint stability. Each muscle contains subregions with distinct anatomical and biomechanical properties, influencing their roles in movement and susceptibility to injury. Muscle activation patterns in various overhead sports highlight the interplay between stabilizers and movers, optimizing performance and minimizing injury risk. In baseball pitching, the SSP, ISP, and SSC stabilize the glenohumeral joint, demonstrating high activation during explosive phases such as stride, arm cocking, arm acceleration, and deceleration [8]. Similarly, tennis serving involves the pectoralis major and latissimus dorsi for power generation, with the RC muscles stabilizing against large rotational forces to support dynamic coordination and injury prevention [8]. Swimming relies on the SSC and latissimus dorsi for propulsion, while volleyball spikes engage the deltoid and SSP for upward arm motion, with the RC muscles stabilizing the shoulder to prevent impingement [8]. Across these sports, effective coordination between stabilizers and movers is critical for biomechanical efficiency and injury prevention. Variations in biomechanical properties and activation among RC subregions significantly influence shoulder mechanics during different motions. These distinctions have vital implications for diagnosing and managing age- or overuse-related pathologies such as RC tears, impingement, and instability. A deeper understanding of RC subregions could enhance diagnostic precision and inform tailored treatment strategies

### Future Research Directions

Despite significant advances, several areas in shoulder biomechanics remain underexplored. While subregions such as the middle ISP and anterior SSP have been well studied, others, like the SSC subregions, lack comprehensive evaluation, particularly during dynamic movements such as throwing or lifting. Additionally, the current limitations of shear wave elastography (SWE) in assessing SSC properties—due to its anatomical location and the inaccessibility of the region to ultrasound—highlight a critical gap in our ability to study this muscle effectively. On the other hand, expanding SWE assessments to include multi-plane tasks and varying loads on other RC muscles would offer valuable insights into real-world biomechanics. Developing standardized protocols for these assessments is essential to ensure consistency and reproducibility across studies.

Addressing the functional roles of each subregion may enable the development of tailored rehabilitation programs and more precise surgical techniques. Advances in imaging and biomechanical analysis could further support personalized treatment protocols based on subregional anatomy, leading to improved patient outcomes and faster recovery. The integration of empirical data with computational musculoskeletal models holds promise for predicting functional outcomes following RC injuries or surgical interventions. Additionally, task-specific analyses, such as overhead throwing or weightlifting, could clarify subregion contributions to force generation and joint stability under various conditions. Understanding these intricate dynamics is critical to advancing the diagnosis and treatment of shoulder pathologies. This approach bridges current knowledge gaps and sets the stage for transformative improvements in clinical care.

## Figures and Tables

**Table 2:** Tensile properties of the RC muscles and subregions [mean (SD)].

Muscle		Ultimate Load (N)	Ultimate Stress (MPa)	Elastic Modulus (MPa)	Stiffness (N/mm)
Infraspinatus [39]					
hanging-arm position	Superior	501 (n.a.)	14.6 (7.7)		109.3 (37.0)
	Mid-superior	662.4 (223.4) **^a^**	25 (n.a.)		142.8 (39.6) **^b^**
	Mid-inferior	330.8 (205.8)	13.0 (6.3)		84.8 (34.6)
	Inferior	716.6 (252.7) **^a^**	30.4 (14.4) **^c^**		169.0 (44.2) **^d^**
60° abduction	Superior	424.4 (154.4)	17.7 (7.6)		158.8 (30.9)
	Mid-superior	696.8 (258.1) **^e^**	29.4 (8.2) **^f^**		205.0 (60.3) **^g^**
	Mid-inferior	301.6 (168.5)	18.0 (8.8)		108.4 (45.5)
	Inferior	406.9 (239.0)	14.0 (6.7)		128.1 (50.6)
Combined (hanging + 60°)	Superior			120 (53.1)	
	Mid-superior			156.8 (56.9) **^h^**	
	Mid-inferior			113.3 (45.9)	
	Inferior			140 (n.a.)	
**^a^**: *p* < 0.003 compared to mid-inferior; **^b^**: *p* < 0.001 compared to superior and mid-inferior; **^c^**: *p* < 0.005 compared to superior and mid-inferior; **^d^**: *p* < 0.001 compared to superior and mid-inferior; **^e^**: *p* < 0.001 compared to superior, mid inferior, and inferior; **^f^**: *p* < 0.002 compared to superior, mid inferior, and inferior; **^g^**: *p* < 0.001 compared to superior, mid inferior, and inferior; **^h^**^:^ *p* < 0.003 compared to superior and mid-inferior.					
Teres Minor (combined outcomes from hanging-arm position and 60° abduction)					
		66.8 (31.0)	1.5 (0.9)	14.1 (9.3)	22.6 (14.4)
Supraspinatus (45° relative to the humeral shaft axis) [40]					
	Anterior	779.2 (218.9) **^b^**	22.1 (5.4) **^c^**	592.4 (237.4) **^a^**	
	Posterior	335.6 (164.0)	11.6 (5.3)	217.7 (102.1)	
**^a^**: *p* < 0.01 compared to posterior; **^b^**: *p* < 0.003 compared to posterior; **^c^**: *p* < 0.008 compared to posterior.					
Subscapularis [46]					
Hanging-arm position	Superior	623 (n.a.)			147.2 (32.3)
	Mid-superior	706 (n.a.)			175 (n.a.)
	Mid-inferior	454 (n.a.)			128 (n.a.)
	Inferior	75 (n.a.)			27.4 (17.7)
60° abduction	Superior	478 (n.a.)			208.7 (60.9)
	Mid-superior	598 (n.a.)			182 (n.a.)
	Mid-inferior	400 (n.a.)			130 (n.a.)
	Inferior	30 (n.a.)			9.5 (5.9)
n.a.: data not explicitly presented in reference					
